# Lightweight and Compact Pulse Radar for UAV Platforms for Mid-Air Collision Avoidance

**DOI:** 10.3390/s25237392

**Published:** 2025-12-04

**Authors:** Dawid Sysak, Arkadiusz Byndas, Tomasz Karas, Grzegorz Jaromi

**Affiliations:** 1 Faculty of Electronics, Photonics and Microsystems, Wroclaw University of Science and Technology, 50-370 Wroclaw, Poland; 2 EUROTECH Ltd., 39-300 Mielec, Poland

**Keywords:** airborne pulse radar, collision avoidance, BVLOS, Sense and Avoid, UAV navigation, obstacle sensing

## Abstract

Small and medium Unmanned Aerial Vehicles (UAVs) are commonly equipped with diverse sensors for situational awareness, including cameras, Frequency-Modulated Continuous-Wave (FMCW) radars, Light Detection and Ranging (LiDAR) systems, and ultrasonic sensors. However, optical systems are constrained by adverse weather and darkness, while the limited detection range of compact FMCW radars-typically a few hundred meters-is often insufficient for higher-speed UAVs, particularly those operating Beyond Visual Line of Sight (BVLOS). This paper presents a Collision Avoidance System (CAS) based on a lightweight pulse radar, targeting medium UAV platforms (10–300 kg MTOM) where installing large, nose-mounted radars is impractical. The system is designed for obstacle detection at ranges of 1–3 km, directly addressing the standoff distance limitations of conventional sensors. Beyond its primary sensing function, the pulse architecture offers several operational advantages. Its lower time-averaged power also results in a reduced electromagnetic footprint, mitigating interference and supporting emission-control objectives. Furthermore, pulse radar offers greater robustness against interference in dense electromagnetic environments and lower power consumption, both of which directly enhance UAV operational endurance. Field tests demonstrated a one-to-one correspondence between visually identified objects and radar detections across 1–3 km, with $P_{\mathrm{FA}}$ = 1.5%, confirming adequate standoff for tens of seconds of maneuvering time, with range resolution of 3.75 m and average system power below 80 W.

## 1. Introduction

In recent years, the use of Unmanned Aerial Vehicles (UAVs) has expanded significantly across civil and military domains, with Beyond Visual Line of Sight (BVLOS) operations receiving particular attention. These flights are typically conducted at low altitudes, often below 120 m (400 feet) Above Ground Level (AGL), which creates a high risk of collision. Obstacles include not only fixed infrastructure, such as buildings, wind turbines, and power lines, but also other low-altitude airspace users, including UAVs and avifauna. This risk is further exacerbated in adverse weather conditions, such as fog and rain, or during nighttime operations, where visibility is limited. Such conditions render optical sensors like cameras ineffective, necessitating the adoption of radar-based technologies that can ensure operational safety regardless of visibility [1].

The urgent need for advanced detection systems is compounded by the dynamic growth [2] of unmanned traffic in airspace, primarily driven by the package delivery sector [3]. The increasing density of operations in urban areas raises the likelihood of incidents, highlighting the necessity for implementing reliable Sense and Avoid technologies [4]. In response to these challenges, this paper proposes a novel Collision Avoidance System (CAS) based on a lightweight pulse radar. This system is designed to achieve a significantly greater detection range of 1–3 km, directly addressing the standoff distance limitations of conventional FMCW radars. Furthermore, its lower average power consumption and smaller electromagnetic footprint translate to longer flight endurance and greater resilience to interference [5]. This approach is preferred when many radars operate in close proximity; additionally, the use of diverse transmission patterns can further enhance that resilience. The proposed system utilizes a miniaturized architecture based on a single Field Programmable Gate Array (FPGA), enabling a balance between performance, weight, and cost.

Building on these findings, we propose a hybrid Sense and Avoid architecture that fuses conventional short-range sensors to cover the UAV’s immediate vicinity (up to 1 km) with a medium-range pulsed radar. The short-range layer provides dense situational awareness and obstacle coverage around the platform, while the pulsed radar extends the detection horizon to enable early warning of more distant hazards along the direction of travel. This layered fusion is particularly beneficial for higher-speed UAVs, where longer look-ahead times are critical for safe trajectory planning and timely avoidance maneuvers. In addition, the proximal short-range sensors are better suited to handling small-RCS, highly maneuverable targets such as birds, especially in urban or cluttered environments, complementing the pulsed radar’s role in earlier, forward-looking detection of larger or more distant obstacles.

Despite extensive research on sensor-based Collision Avoidance Systems (CAS), current solutions still leave a critical gap between short-range, low-power sensors and large, high-performance airborne radars. Existing lightweight FMCW and LiDAR-based systems typically provide only a few hundred meters of effective detection range, while long-range pulse and hybrid radars reported in the literature have not closed the range–power–form-factor trade-off required for medium UAV platforms and BVLOS operations. Motivated by these limitations, this work introduces an X-band pulse radar architecture that targets 1–3 km standoff detection with a controlled false-alarm probability, average power below 80 W, and a compact, nose-mountable form factor. The paper demonstrates, through detailed hardware design and field experiments in representative urban and open-air scenarios, that such a medium-range CAS radar can be realized within realistic constraints on size, mass, and power consumption, thereby providing a practical sensing layer between conventional short-range sensors and large airborne surveillance radars.

The paper is structured as follows: Section 2 analyzes existing collision avoidance methods. Section 3 describes the architecture of the proposed radar sensor. Section 4 outlines the test methodology, and Section 5 presents and discusses the results. The paper concludes with a discussion and summary in Section 6 and Section 7.

## 2. Review of Current Collision Avoidance Methods

The advancement of UAV technology requires the development of effective obstacle detection and CAS, particularly for operations in urban environments and adverse weather conditions. Current methodologies can be classified into several main categories, each with distinct advantages and limitations [5].

The first category consists of systems based on electro-optical sensors, such as cameras operating in the visible spectrum (approximately 380–750 nm) and in infrared windows (e.g., 1–15 μm). Cameras, commonly used on UAVs, offer high spatial resolution and object recognition under favorable illumination conditions [6]. However, their effectiveness deteriorates rapidly in fog, rain, or darkness [1], limiting their applicability in dynamic scenarios [7]. Moreover, vision-based systems also require significant onboard data processing, imposing a substantial computational load and increasing energy consumption.

The second category comprises LiDAR (Light Detection and Ranging) systems, which use laser pulses to create precise, three-dimensional maps of the surrounding environment [8]. Their key advantage is the ability to deliver highly accurate spatial data independent of ambient light, which is valuable for advanced mapping applications. However, multi-beam LiDAR scanners designed for small and medium-sized drones must trade off cost, weight, and performance; consequently, many lightweight models [9,10] have an effective range limited to approximately 100–300 m. Reported LiDAR ranges are highly scenario-dependent due to atmospheric attenuation and backscatter. Recent surveys review these weather-driven limitations [11]. Beyond mapping applications, modern single-point LiDAR sensors [12] can achieve ranges in excess of 1 km, typically for high-albedo targets under favorable atmospheric conditions. Although these sensors present a potential solution for CAS on small UAVs, such ranges remain insufficient for advanced applications requiring detection out to roughly 3 km. Furthermore, all LiDAR systems, performance degrades significantly in adverse atmospheric conditions, which remains a fundamental limitation.

The third category of sensors is radar, notably FMCW systems. These are particularly valuable because they enable obstacle detection largely regardless of weather conditions, a significant advantage over optical and LiDAR sensors [11,13]. FMCW radars provide continuous transmission that enables simultaneous range and radial velocity measurements [14]. This capability, combined with relatively simple hardware and lower peak power compared to pulse systems, makes them suitable for UAV integration. However, lightweight, low-power implementations for small to medium size UAVs often have limited detection range-typically capped at a few hundred meters-which is insufficient for faster UAVs operating over longer distances or for detect-and-avoid scenarios with high closing speeds. Finally, while individual FMCW systems can be low power, extending range generally necessitates higher transmit power, which in turn negatively impacts the flight endurance of battery-powered UAVs.

Other methods, such as ultrasonic sensing, are common in low-cost UAVs [15,16] but are generally limited to 10–20 m line-of-sight and are susceptible to multipath and environmental noise, rendering them unsuitable at higher altitudes or in cluttered urban environments. Sensor fusion combining cameras, LiDAR, and radar seeks to mitigate individual weaknesses; however, system complexity and computational load remain significant challenges [5].

Other documented attempts to build CAS for drones rely on pulse radars or hybrid solutions (pulse with FMCW/CW elements), typically with electronic scanning to meet coverage requirements. As shown by works [17,18], the X band offers a favorable trade-off in terms of angular resolution, antenna size, and integration cost. Unfortunately, with a required warning time on the order of several tens of seconds (e.g., 20 s), which translates to ranges of about 2–3 km, the described implementations do not close the energy and/or scanning budget within an acceptable mass and volume form factor [19] for small and many medium UAVs. In particular, the lightweight X-band radar described in [18] offers a range of about 70 m, which is an order of magnitude too low relative to CAS operational requirements.

In light of evolving operational requirements-growing drone density in cities and emerging applications, including package delivery and public safety operations, current methods exhibit clear limitations. This motivates the development of lightweight autonomous sensing solutions, such as pulse radars, offering greater range, a lower electromagnetic signature, and improved compatibility with standards such as NATO STANAG 4671 [20]. The subsequent sections present the detailed design and testing of the proposed solution.

**Figure 1:**
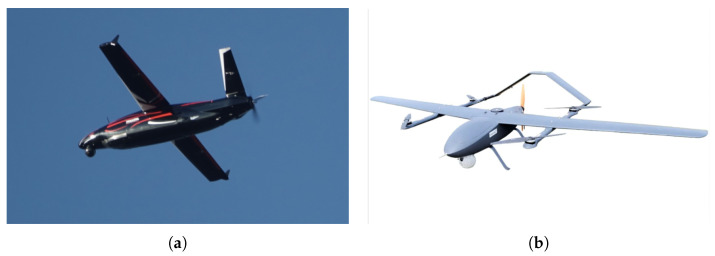
(**a**) The HAASTA UAV, with a wingspan of 3.89 m and a payload capacity of up to 30 kg (source: eurotech.com.pl). (**b**) The CK 23VE C6-class UAV, featuring a 3.2 m wingspan and an MTOM of 25 kg (source: aeroexpo.online).

The above review frames the research objective: the development of a pulse radar sensor for BVLOS operations and air-situation awareness at standoff distances of several kilometers. UAVs capable of carrying a lightweight pulse radar include aircraft in the C5 and C6 certification categories, as well as professional and industrial grade platforms, as illustrated in Figure 1.

To provide a comprehensive context for the performance of our proposed pulse radar system, we have included a comparative analysis of recent relevant UAV radar systems reported in the literature, alongside representative commercial solutions (see Table 1).

## 3. Radar Description

### 3.1. Design and Functional Overview

**Figure 2:**
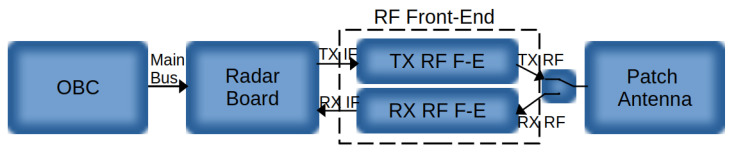
Block diagram of the main modules of the radar sensor.

The proposed radar sensor comprises four principal modules, as illustrated in Figure 2: The On-Board Computer (OBC), the Radar Board, the RF Front-End (including TX and RX Front-Ends), and the Radar Antenna. To minimize mass and complexity, the envisioned implementation places a lightweight, cost-effective, fixed forward-looking radar in the aircraft nose, avoiding any gimballed actuation while focusing solely along the flight axis. A detailed description of each module is provided below.

The OBC installed on the aircraft serves as the primary processing and control unit for multiple avionics systems. It acquires and manages sensor data, executes real-time processing algorithms, coordinates system operations, and provides communication with both avionics subsystems and ground control stations. The OBC also monitors system health, manages timing and synchronization tasks, and supports both autonomous and pilot-assisted operations. For laboratory testing, the radar sensor can alternatively be interfaced with a personal computer, enabling accelerated debugging, calibration, and evaluation without the UAV avionics layer.

**Figure 3:**
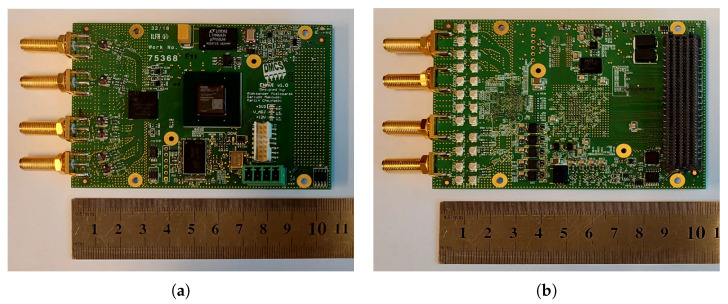
(**a**) Radar PCB top layer. (**b**) Radar PCB bottom layer. PCB of the radar module, board dimensions: 105 × 70 mm. Weight: 60 g.

The Radar Board, developed within the framework of project IDAAS (see Section 4) is a single printed circuit board (PCB) measuring 105 × 70 mm, as shown in Figure 3. This technologically advanced board incorporates several key components, with the Direct IF transceiver and the FPGA as its centerpieces.

The transceiver is a fully programmable wideband intermediate frequency (IF) device based on the LMS7002 chip [25]. It provides two independent full-duplex TX/RX channels, supporting operation in both MIMO and time-division duplexing modes. Its architecture employs direct conversion (zero-IF), which substantially simplifies the signal chain by eliminating the need for multiple IF stages, filters, or amplifiers operating at different frequencies. Analog signals are directly converted to and from digital I/Q base-band signals. The modulated IF signal can be expressed as:

(1) $$s\left(t\right)=I\left(t\right)\cdot \cos\left(2\pi f_{c}t\right)-Q\left(t\right)\cdot \sin\left(2\pi f_{c}t\right)$$

or equivalently:

(2) $$s\left(t\right)=A\left(t\right)\cdot cos(2\pi f_{c}t+\varphi \left(t\right))$$

where s(t) is the analog IF signal, I(t) and Q(t) are the in-phase and quadrature components, $f_{c}$ is the carrier frequency, $A\left(t\right)=\sqrt{I^{2}\left(t\right)+Q^{2}\left(t\right)}$ is the magnitude of the I/Q vector, and φ(t) is the phase of the I/Q vector. The transceiver operates over a continuous frequency range starting from several hundred kilohertz up to nearly 4 GHz, enabling its use across diverse radio communication standards, from shortwave to microwave bands. Each RF path is equipped with 12-bit analog-to-digital (ADC) and digital-to-analog converters (DAC), supporting instantaneous bandwidths up to 160 MHz. The chip integrates comprehensive analog and digital filtering blocks as well as advanced digital signal processing capabilities, facilitating flexible adaptation to various protocols and dynamic radio parameter tuning. This device also features advanced automatic calibration routines, an embedded micro-controller, and a digital interface compliant with the communication standards of contemporary wireless systems, suitable for both prototype development and production deployment. Despite its broad functionality, the transceiver maintains relatively low power consumption, making it well-suited for mobile and battery-powered applications. The high level of integration substantially reduces device complexity and cost, while accelerating system design and implementation cycles. As of 2025, this transceiver is commercially available through multiple electronics distributors.

The FPGA [26] serves as the core of the signal processing subsystem in the radar. Its primary responsibilities include the initialization and real-time calibration of the Intermediate Frequency (IF) transceiver, as well as precise timing control for the transmit/receive (TX/RX) keying of the RF signal. Configuration of the IF stage is accomplished via a Serial Peripheral Interface (SPI). For high-throughput data transfer, digitized 12-bit TX and RX signals are exchanged across two independent Double Data Rate (DDR) memory interfaces. An efficient pulse compression algorithm, based on FFT correlation with a pipelined architecture, is implemented within the FPGA. Finally, the generated radar plots are streamed to the On-Board Computer (OBC) for higher-level processing.

The RF Front-End is composed of two primary subsystems: the TX Front-End and the RX Front-End. The TX Front-End is responsible for the up-conversion of the intermediate frequency signals from the 1–3 GHz range to approximately 9 GHz, corresponding to the X-band. In our system, the frequency translation process proceeds as follows.

The incoming IF signal, typically between 1 and 3 GHz, is first passed through a band-pass filter designed to suppress out-of-band noise and spurious signals. The output power of the IF signal is controlled by a variable gain amplifier integrated within the IF transceiver chip, enabling precise adjustment of signal levels before up-conversion.

The Local Oscillator (LO) chain plays a critical role in generating and distributing a clean, stable reference frequency to all mixers in both the RX and TX paths. Proper LO design ensures high spectral purity with low phase noise and minimal spurious emissions, provides sufficient drive power, and maintains excellent isolation. These factors are essential to achieve low distortion, reduce signal leakage, and optimize overall system performance.

At the core of the up-conversion stage is the frequency mixer, a non-linear component that combines the IF input signal with the LO signal. The mixer generates components at $f_{L}O\pm f_{I}F$, where $f_{LO}$ is the LO frequency, and $f_{IF}$ is the IF signal frequency. Following mixing, the output contains the desired component along with an image component (the opposite sideband relative to the LO), as well as a certain level of LO leakage and additional spurious products from device nonlinearity. To isolate the desired RF band near 9 GHz, highly selective band-pass cavity filters are employed to suppress the image, harmonics, and other spurious signals.

Subsequently, the filtered RF signal is amplified through two stages of power amplification, achieving a final output power level of approximately 43 dBm. Power amplifiers, especially the final stage, generate significant heat. Therefore, precise control circuitry is implemented to enable the shutdown of the amplifier between transmissions and adequate thermal management solutions are employed to maintain safe operating temperatures.

The next critical element in the RF front-end is the duplexer, commonly realized as either a switch or circulator. This component multiplexes the antenna port alternately between the transmit (TX) and receive (RX) paths, allowing a single antenna to be shared for both signal transmission and reception.

Alternatively, the system architecture may employ two separate antennas—one dedicated exclusively to transmission and the other to reception. This dual-antenna approach presents distinct advantages and disadvantages. The advantages of separate TX and RX antennas include improved isolation by at least 15 dB, as physical separation reduces direct coupling and self-interference between transmit and receive signals, thus enhancing receiver sensitivity and dynamic range. Reduced insertion loss is another benefit, as eliminating the need for a switching element or circulator reduces insertion loss in the RF path, improving the overall system link budget and power efficiency. Moreover, dedicated antenna chains allow for independent optimization of impedance matching and filtering in TX and RX paths, simplifying signal routing.

The disadvantages of using separate TX and RX antennas involve increased system complexity and size, since the incorporation of two antennas increases physical footprint, weight, and mechanical complexity, which can pose constraints in airborne or compact platforms. Additionally, different antenna radiation patterns and phase centers can complicate antenna pattern calibration, particularly in phased array or beamforming applications.

In contrast, the use of a duplexer sharing a single-antenna port is more compact and cost-effective but may introduce concerns such as insertion loss, limited isolation between TX and RX paths, and potential nonlinearities under high transmit power conditions. The choice between these configurations depends on application-specific trade-offs involving size constraints, system complexity, desired performance, and cost considerations.

Throughout the RF front end, meticulous impedance matching is maintained at 50 Ω to ensure maximum power transfer and minimal signal reflection, thus optimizing overall system efficiency. The RX front-end comprises modules similar to those found in the TX path. However, there are several critical differences that must be highlighted. At the input of the RX chain, a limiter is employed to protect sensitive amplifier stages from excessive input power, thereby safeguarding the Low Noise Amplifier (LNA) and subsequent circuits from damage due to high-power signals or signal surges. Following the limiter, the signal passes through a network of LNAs, which provide essential low-noise gain to boost weak received signals while minimizing the addition of noise. The amplified signal is then processed by cavity band-pass filters. These high-selectivity cavity filters suppress unwanted frequencies and out-of-band interferences, ensuring that only the desired signal band is delivered to the subsequent stages.

The filtered and amplified signal is then routed to the mixer, which, in the receive path, operates as a down-converter. In this stage, the incoming RF signal is translated down to an IF by mixing it with a LO signal. This frequency conversion step is essential for further signal processing at lower, more manageable frequencies. After down-conversion, the resulting IF signal is passed through additional band-pass filters tailored to the intermediate frequency. These filters further suppress spurious products, image frequencies, and other undesired spectral components arising from the mixing process, thereby ensuring that a clean IF signal is delivered for subsequent demodulation.

The antenna presented in this study is a planar, dual-polarized patch array designed to meet the stringent requirements characteristic of contemporary radar systems, particularly those demanding high gain, thin profile, low mass, and multi-polarization operation. The array, as described in [27] is based on a modular architecture, where each tile serves as a self-contained 4 × 4 element subarray constructed using advanced multilayer sandwich technology. Each tile comprises up to 20 bonded layers—including low-loss laminates, foams, and bonding films—which ensures structural rigidity and electrical stability, while maintaining a total thickness below 10 mm per module.

The integration of encapsulated printed stripline circuits within these multilayer modules enables efficient power distribution to each antenna element. To further minimize transmission losses and maximize array efficiency, the modules are coupled via custom-designed rectangular waveguide power splitters. These waveguides, fabricated using a combination of precision milling, metal casting, and additive manufacturing techniques, exhibit non-standard dimensions (20 × 8 mm) specifically chosen to reduce the overall antenna profile while supporting high power-handling capacity and low insertion loss.

**Figure 4:**
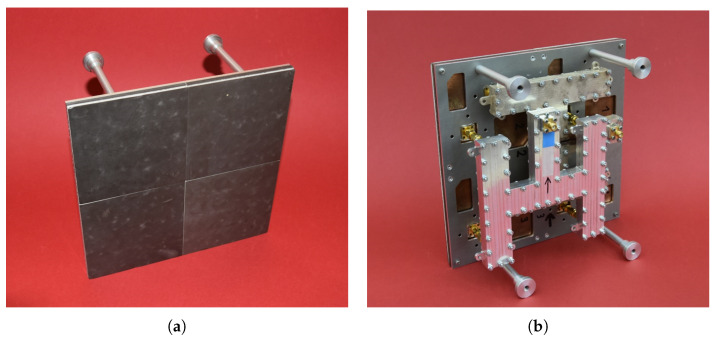
X-band radar antenna: (**a**) front view, (**b**) rear view, the arrow indicates the direction of polarization. Aperture dimensions: 200 × 200 mm. Weight: 500 g.

The complete antenna array, shown in Figure 4, consists of four such tiles arranged in an 8 × 8 element matrix, yielding a compact form factor of 20 × 20 × 4 cm. This design achieves measured gains up to 27.4 dBi for both horizontal and vertical linear polarizations, with an operational bandwidth exceeding 1 GHz in the X-band. Dual polarization is implemented via two fully isolated feed networks, one per polarization, improving inter-polar isolation and minimizing mutual coupling. The array was developed within the IDAAS project framework (see Section 4).

**Figure 5:**
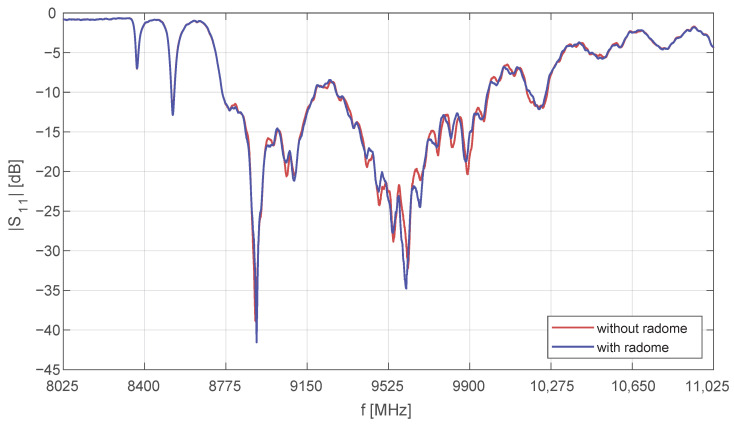
Measured input return loss (S11) of the X-band radar antenna. The radome is made of fiberglass and coated with paint.

The antenna measurement results presented in Figure 5 demonstrate excellent return loss and impedance matching across the intended frequency range, with the lowest loss contributions observed in the multilayer stripline segments and the waveguide distribution network (total loss < 0.7 dB; system efficiency ≈ 85%).

**Figure 6:**
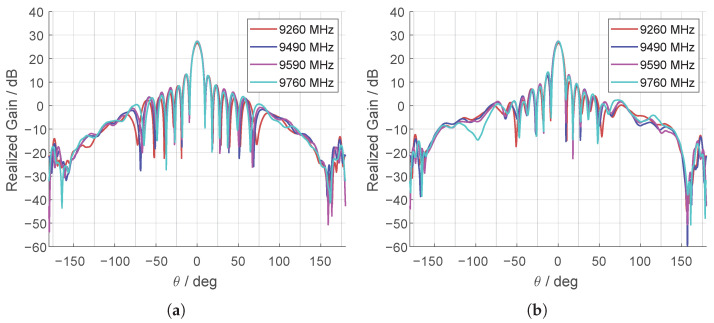
Measured radiation patterns of $E_{\theta }$ for the co-polar component of the 64-element antenna: (**a**) $E_{\theta }\left(\theta ,\phi =0{}^{\circ}\right)$, (**b**) $E_{\theta }\left(\theta ,\phi =90{}^{\circ}\right)$.

The radiation patterns shown in Figure 6 exhibit a narrow directive main beam and low sidelobe levels for both co-polar and cross-polar components, confirming their suitability for high-resolution radar applications. Beyond its performance metrics, the presented architecture allows scalable adaptation to more complex beamforming systems, including AESA and MIMO radar arrays. The dual-polarization support and thin profile make it highly effective for installation in airborne, shipborne, and ground-based radar platforms—especially where conformal or space-limited configurations are desirable.

The antenna can be considered as an interchangeable element depending on the specific requirements of the UAV carrier. The antenna proposed here is only an example and its size is certainly too large for platforms with an MTOM of 25 kg. For smaller UAVs, it is possible to install a smaller antenna, e.g., a single module from the antenna shown in Figure 4, with dimensions 10 × 10 cm.

**Figure 7:**
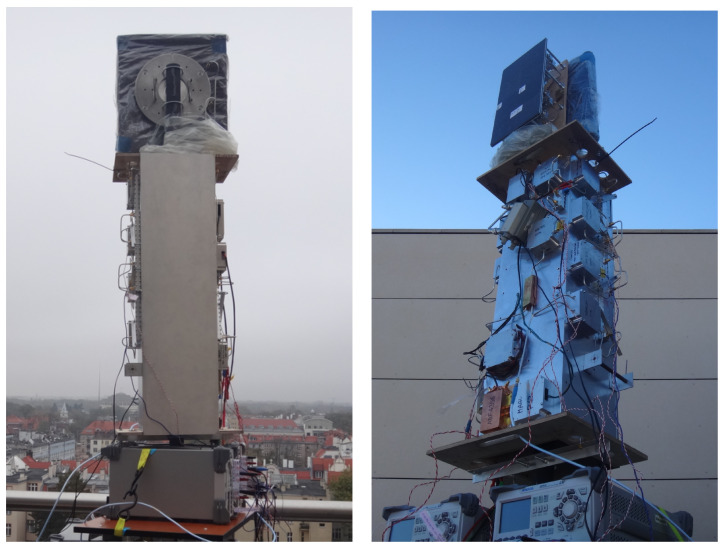
Experimental version of the radar used during measurements. The images show the radar antennas and microwave circuits. Other subsystems are outside the image frame.

The overall system dimensions and mass largely depend on the RF front-end module, which currently relies on development hardware temporarily mounted on the rectangular metal frame shown in Figure 7. The indicative dimensions of this module are approximately 80 × 30 × 30 cm, with a mass of about 4 kg (excluding the power supply). The standard antenna measures 200 × 200 mm and weighs about 500 g. The digital radar board measures 105 × 70 mm and weighs 60 g.

### 3.2. Radar Parameters

The radar is a flexible, firmware-defined system that supports operation across a wide range of waveform and processing parameters. Typically, experiments were conducted using the default configuration summarized in Table 2, which ensures reproducible baseline performance and consistent comparison across test runs. At the same time, the radar allows rapid reconfiguration of key settings to match mission objectives and adapt to changing environmental conditions, such as varying target ranges, clutter levels, and interference.

The power reported in Table 2 refers to the average power draw under default settings. This value varies with the radar’s configurable parameters. For primary operating modes, the average power consumption is: 78 W (1–3 km, high PRF); 72 W (1–3 km, default PRF); 68 W (1–3 km, low PRF); and 77 W (3–7 km, long-range, default PRF).

Table 3 lists the parameters that can be reconfigured at runtime, including waveform type and bandwidth, pulse duration, pulse repetition frequency (PRF), number of coherent integrations, receive gain, and detection thresholds. These parameters can be updated without restarting the system, enabling quick trade-offs between range resolution, maximum unambiguous range/velocity, and sensitivity. The control interface updates parameters in a single transaction to prevent transient misalignment between the transmit and receive paths. In practice, the full parameter set can be updated within a few milliseconds, which supports agile retasking (e.g., switching between high-resolution and medium-range modes) and closed-loop adaptation driven by on-board environment and performance monitors. This responsiveness is particularly beneficial in dynamic scenarios, where rapid transitions between modes minimize dwell time penalties and maintain detection performance under non-stationary conditions.

Modern radar target detection hinges on adaptive thresholding to ensure reliable performance under spatially and temporally varying noise and clutter conditions; among these techniques, Constant False Alarm Rate (CFAR) methods remain foundational due to their balance between detection sensitivity and controllable false-alarm probability. In recent decades [28,29,30,31], numerous CFAR detectors have been proposed, including classical cell-averaging (CA-CFAR), greatest-of (GO-CFAR), smallest-of (SO-CFAR), as well as ordered-statistics (OS-CFAR) and their numerous extensions, enabling robust target detection in the presence of heterogeneous clutter, interfering targets, and varying noise conditions.

**Figure 8:**
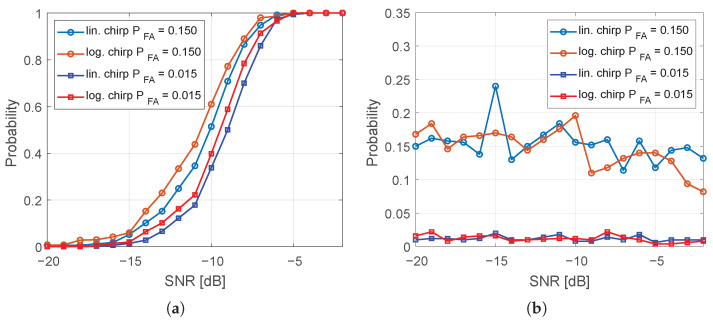
(**a**) Probability of target detection ($P_{D}$). (**b**) Probability of false alarm ($P_{\mathrm{FA}}$).

The proposed radar system adopts a CA-CFAR detector. An estimation of the probability of detection ($P_{D}$) and probability of false alarm ($P_{\mathrm{FA}}$) was conducted, as shown in Figure 8. The procedure described in [32] involved transmitting a chirp signal through a 30 μs optical delay line. To vary the signal-to-noise ratio, a variable attenuator was applied to the transmit output of the radar sensor. Two threshold detection algorithms were employed. The first algorithm set the probability of false alarm at 15%, while the second algorithm reduced this parameter to 1.5%. As expected, a higher $P_{\mathrm{FA}}$ results in an increased probability of detection; however, this comes at the cost of a higher susceptibility to false alarms. Our configuration follows contemporary treatments of detection under clutter for mobile platforms, complementing classical CFAR analyses with recent advances [31,33].

Furthermore, Figure 8 presents a comparison between two types of probing signals: a linear chirp and a logarithmic chirp. The tests indicate that the logarithmic chirp tends to achieve higher $P_{D}$ at similar $P_{\mathrm{FA}}$ levels.

## 4. Experimental Methodology for Radar Performance Evaluation in Urban Scenarios

**Figure 9:**
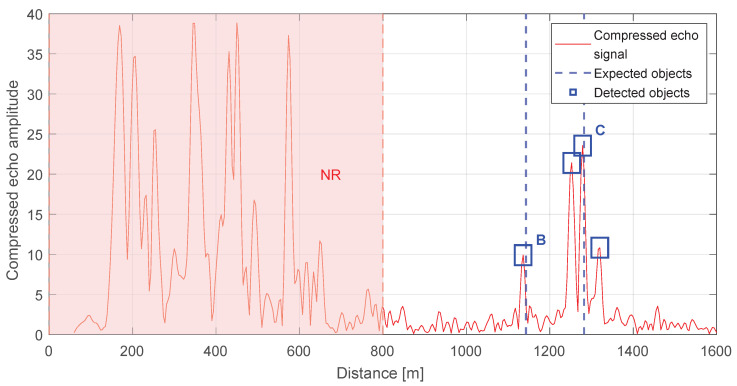
The range-radar output over 0–1.5 km with annotations for potential obstacles and detections. The annotations (NR, B, C) are defined in Table 4.

For the empirical validation of the object detection performance of the radar presented in Section 3, a series of field tests was performed in a representative urban environment. These evaluations utilized the ENAVI radar [34,35], a system resulting from a collaborative effort between our research team and EUROTECH Ltd., developed within the scope of the Intruder Detection And Collision Avoidance System (IDAAS) project. The experimental campaign focused primarily on the detection and characterization of large static urban objects, such as residential buildings and tall architectural landmarks, which served as stable high-RCS references for assessing detection performance and calibration stability. The selected objects were positioned at medium distances, ranging from 1 to 3 km from the radar site. The objects positioned within the Near Range (NR) zone (nearer than 800 m) were excluded by design as shown in Figure 9 because such ranges are outside the focus established in prior sections, which additionally limits multipath and near-range clutter.

All measurements were conducted from an elevated observation point—a terrace of building D-20, approximately 35 m above ground level—which provided an unobstructed Line of Sight (LOS) towards the selected urban landmarks. The tests focused on detecting objects that may pose potential threats to low-flying UAVs. Measurements were carried out using the radar demonstrator sensor shown in Figure 7, with the default radar parameters listed in Table 2. The antenna was oriented so that several objects, specified in Table 4, were within its beam, enabling the acquisition of representative radar responses from prominent, distant urban targets while minimizing interference from nearby structures and ground-level reflections.

Throughout the remainder of this paper, we adopt the nomenclature summarized in Table 4: (A) denotes the radar position, NR the near-range zone, and (B–G) indicate candidate obstacles for a low-flying UAV. Table 4 reports, for each labeled object, a concise description, geographic coordinates retrieved from the public geodetic portal geoportal.gov.pl, and the distance to point (A) (the scanning position). Distances are computed from the reported coordinates and are intended to serve as ground-truth references for subsequent analysis. The overall distance accuracy is estimated at ±1 m. This budget comprises approximately ±15 cm due to rounding of the GPS coordinates, complemented by the uncertainty in selecting the exact point of interest on the map and minor geolocation discrepancies inherent to map projection and datum transformations. For clarity and reproducibility, all coordinates are reported in WGS-84 latitude/longitude, and distances are expressed relative to (A), which is treated as the fixed scanning origin.

**Figure 10:**
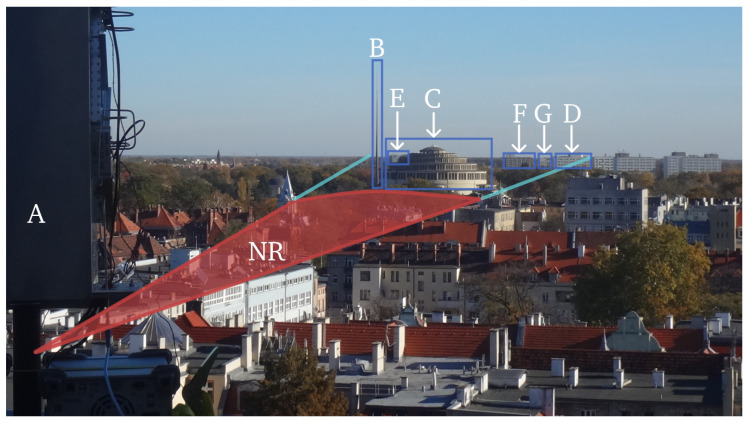
The overview of the scene from the radar’s vantage point. The 3 dB antenna beam has been overlaid, within the NR zone it is marked with a red line, and at farther ranges with a light-blue line. The annotations (NR, A–G) are defined in Table 4.

A visual depiction of the objects listed in Table 4 is provided in Figure 10, rendered from the radar’s vantage point to reflect the sensor’s LOS geometry. The 3 dB contour of the antenna’s main-lobe pattern is overlaid to indicate the effective angular region of high gain, thereby specifying which objects fall within the primary beam during the acquisition. All designations (A–G) are annotated directly on the image to ensure one-to-one correspondence with the identifiers used in Table 4 and the subsequent analysis.

The scene illustrates multiple elevated, static structures distributed over a broad range of distances and apparent sizes, which naturally produces partial mutual occlusions depending on bearing and elevation. This visual arrangement highlights several practical aspects relevant to urban operation: (i) objects at similar azimuths but different ranges can mask one another, (ii) the projected extent of wide facades leads to angle-dependent overlap within the main-lobe footprint, and (iii) small changes in pointing may admit or exclude specific targets at the 3 dB boundary. Together, these effects help explain variations in apparent target strength and detectability reported in later sections.

## 5. Results of Test Campaign

**Figure 11:**
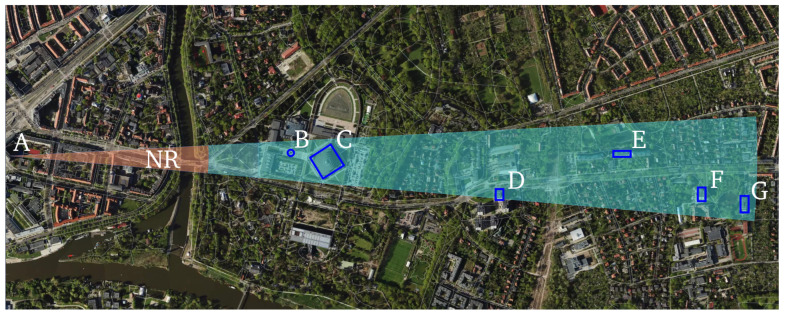
Horizontal projection of the radar scanning field. The red sector denotes the NR zone, while the light-blue sector indicates the range of interest (1–3 km). Detected objects are marked with blue rectangular overlays. The annotations (NR, A–G) are defined in Table 4. Data source: geojson.io.

**Figure 12:**
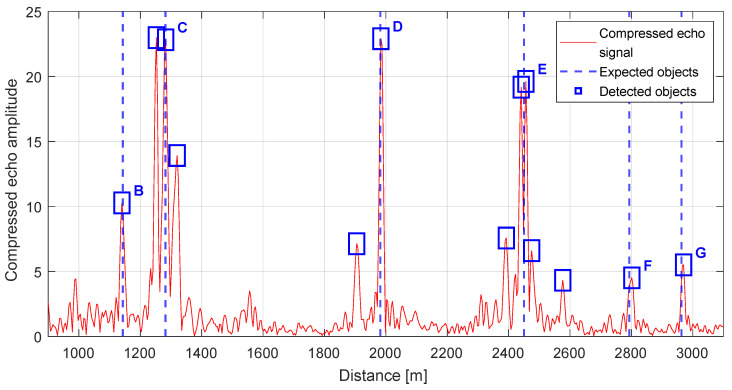
Raw range-radar output over 1–3 km with annotations for potential obstacles and detections. The annotations (B–G) are defined in Table 4, and the complete list of detected objects is provided in Table 5.

The datasets in Figure 11 and Figure 12 comprise scene elements that the radar classified as potential hazards to a low flying UAV, including elevated, static structures within the 1–3 km range of interest. Figure 12 presents the radar return at the output of the range compression stage, shown post-detection to emphasize the distribution of threshold exceedances across range cells.

**Figure 13:**
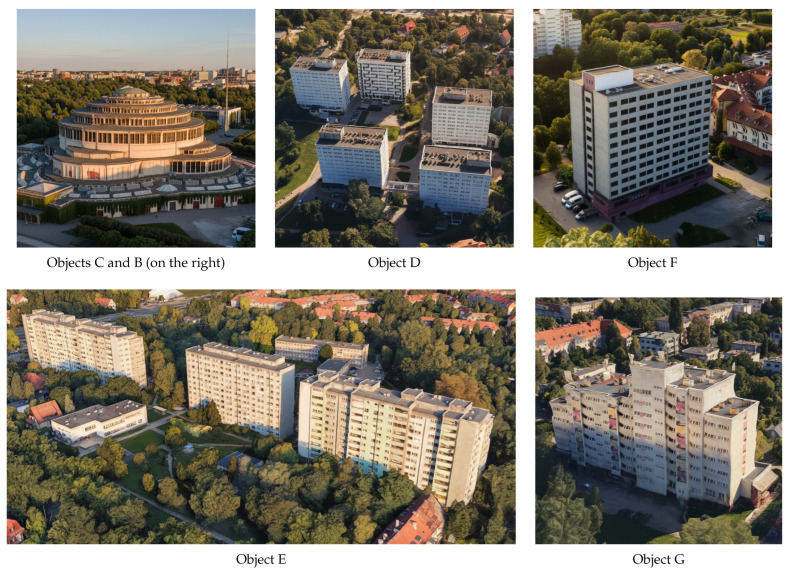
Images of radar-detected objects, corresponding to the entries listed in Table 4. Data source: Google Maps.

Detection is performed using a locally adaptive threshold configured to maintain a constant $P_{\mathrm{FA}}$ of 1.5%. This CFAR strategy stabilizes sensitivity under spatially varying background conditions by estimating noise and clutter statistics over sliding reference windows and modulating the threshold accordingly. Under these settings, all objects visually identified in a co-registered daylight photograph with good visibility were successfully detected by the radar, yielding a one-to-one correspondence between labeled scene elements and radar detections. The photograph is registered to the radar vantage point to preserve line-of-sight geometry, and object labels are shared across modalities (A–G) to enable direct cross-referencing with Table 4 and the plan-view schematic. Figure 13 presents spatial graphical representations for each of the identified objects.

For extended targets like large facades, spatially extended buildings (e.g., (C)—Centennial Hall, detections typically appear as compact clusters near the strongest specular returns, which is consistent with the expected scattering behavior at the given incidence angles. These objects include (C), (D), and (E), which constitute prominent, elevated reflectors within the radar’s field of view. In Figure 12, multiple secondary peaks are evident in the vicinity of the principal echo, forming localized clusters that suggest scattering from adjacent structural features. In the remainder of this section, we undertake a detailed attribution of these smaller responses, examining their consistency across neighboring range cells, their persistence over successive scans, and their alignment with known object geometries and viewing angles.

It is noteworthy that, outside the detection clusters surrounding the candidate objects, the background level remains relatively low and spatially uniform, with no significant out-of-cluster returns. This behavior is consistent with a properly tuned constant false-alarm detector: the adaptive threshold suppresses background excursions while maintaining sensitivity to structurally meaningful scatterers, thereby limiting false positives without compromising detection of operationally relevant targets.

Objects located within the NR zone are automatically ignored and excluded from the processing window. This follows from the deterministically short echo return time of nearby reflectors, which for 800 m corresponds to 5.33 μs. The effect is illustrated in Figure 9, where the radar processing window was intentionally opened earlier than nominal to capture echoes from close-in objects. Under standard operating conditions, the recording start is delayed by a preset offset, thereby suppressing these near-range returns. This strategy additionally enables appropriate gain setting in the receive chain for the intended range interval (in this case, 1–3 km), improving dynamic range utilization and mitigating saturation from strong near-field scatterers.

For diagnostic purposes, we verified that moving the window earlier recovers the expected NR responses with high amplitude and short time-of-flight, confirming the correctness of the timing budget and the efficacy of the gating strategy. In routine operation, the selected delay and gain profile are fixed for a given campaign and documented alongside the calibration parameters, ensuring reproducibility and consistent interpretation of detections within the 1–3 km range of interest.

As noted earlier, objects (C), (D), and (E) exhibit distinct spatial scattering characteristics in the radar output, indicative of their geometry and relative placement within the beam footprint. Table 5 lists all detection points together with their geolocation, the true distance to the corresponding physical structure, and the associated measurement error, enabling direct cross-referencing between the radar domain and the mapped scene.

For object (C), the leading secondary peaks are attributable to the building’s complex geometry, comprising several vertical facades laterally offset by a few meters. These offsets produce multiple specular components at slightly different ranges, yielding a cluster of precursor echoes ahead of the principal return. The observed inter-peak spacing is consistent with the physical separations inferred from the plan view, and the relative amplitudes suggest angle-dependent reflectivity across the staggered facade segments.

**Figure 14:**
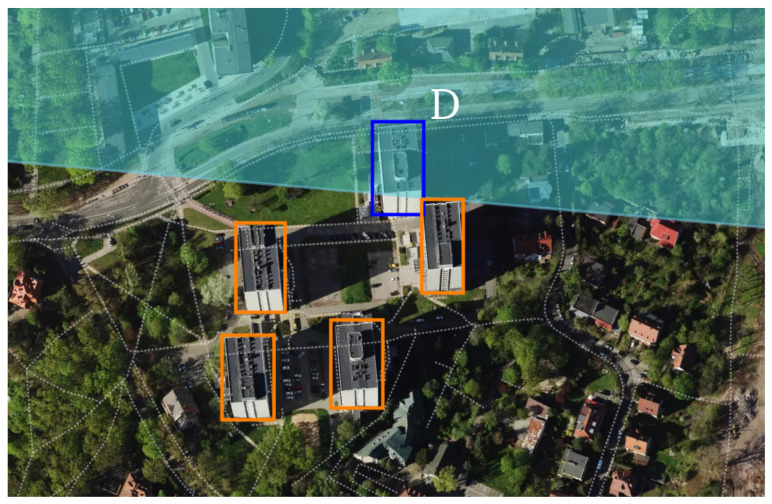
Horizontal projection of the radar scanning field around Dormitory T-22 (object D), reveals several closely spaced, similarly sized buildings. The 3 dB radar beam is indicated in light blue. The principal detected object is marked in dark blue. Adjacent buildings are highlighted in orange, and their returns are attenuated, since they lie outside the main beam. Data source: geojson.io.

For object (D), as shown in Figure 14, the scene consists of a complex of several buildings. The preceding secondary peak originates from the two leftmost buildings, which are located at essentially the same range from the radar. Their reduced echo amplitude stems primarily from their proximity to the edge of the antenna 3 dB beam, as well as from the long cumulative facade extent that fosters interference effects and diminishes the apparent return. The remaining buildings in this cluster extend outside the 3 dB beam and are partially occluded by the nearer structures, resulting in a lack of detections.

**Figure 15:**
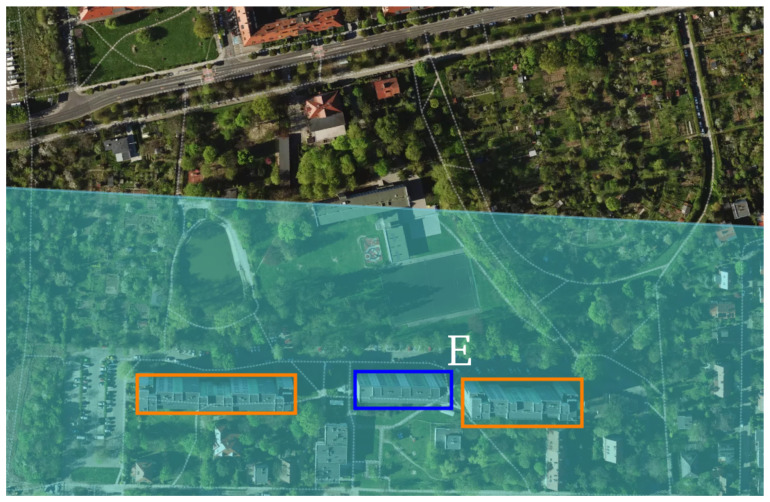
Horizontal projection of the radar scanning field around Residential building at Kazimierska street (object E), reveals several closely spaced, similarly sized buildings. The 3 dB radar beam is indicated in light blue. The principal detected object is marked in dark blue. Adjacent buildings are highlighted in orange, and their returns are attenuated because they are mostly occluded. Data source: geojson.io.

For object (E), visible in Figure 15, a sequence of returns is observed, with the strongest echo arising from the central building. This is because the first building is largely occluded by object (C) as can be observed in Figure 10, leaving only chimneys and limited roof elements exposed, which generate comparatively weak reflections. The main return is preceded by a strong echo from a facade located several meters closer to the radar, itself partially obscured by building (C). The final peak in this group corresponds to a depth offset in the rear facade, consistent with a protruding elevation at the back of the last building, and its timing aligns with the expected additional path length for the recessed structure.

Detailed data for the detected objects, that is, the distance measured using GPS points, the distance derived from the radar, and the corresponding error, are reported in Table 5. For clarity, the dataset is organized into groups associated with the principal radar return for each scene element, enabling direct comparison between the main echo and neighboring secondary peaks. The echoes are indexed from left to right according to their ordering in Figure 12, ensuring consistent cross-referencing between the visualization and the tabulated entries.

### Dynamic-Target Field Tests (Open-Air)

**Figure 16:**
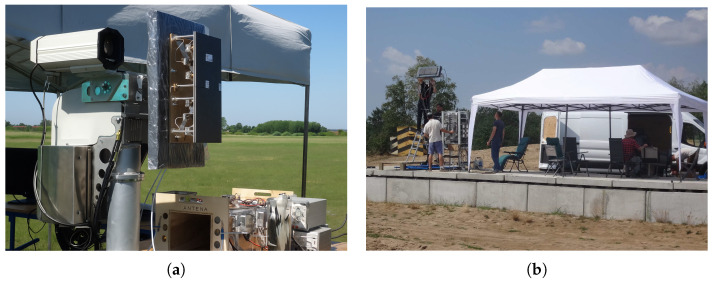
Measurement setups during field tests. (**a**) Small aerodrome in Tuszów during detection tests with a Cessna 172. (**b**) Test ground in southern Poland during detection of the Szogun UAV (wingspan 3.15 m).

**Figure 17:**
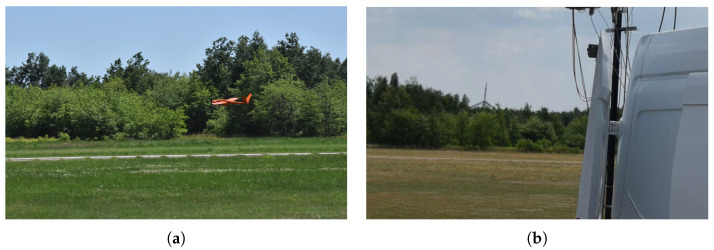
Radar targets. (**a**) Szogun UAV (wingspan 3.15 m) during landing. (**b**) Wind turbine near Grochowe.

Beyond the urban experiments reported above, the authors conducted multiple open-air field tests against dynamic targets to assess moving-object detection capability. This subsection summarizes three representative trials with the same ENAVI radar operated at a different site: (1) a Cessna 172 airplane, (2) a fixed-wing UAV with 3.15 m wingspan and 30 kg MTOM, and (3) a wind turbine. The measurement setups for trials (1) and (2) are shown in Figure 16, while the setup for (3) is presented in Figure 17b; the rotating wind turbine is also visible in the background of that figure. We do not include our own photographs of the well-known Cessna 172 airframe because the aircraft departed from a different aerodrome.

**Figure 18:**
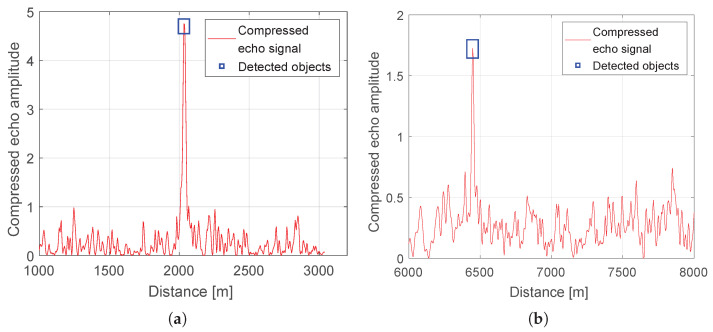
Object detections. (**a**) Wind turbine detected at 2033 m. (**b**) Szogun UAV (3.15 m wingspan) detected at 6447 m.

Figure 18a shows the radar response from a rotating wind turbine located 2025 m away according to GPS markers, while the radar-measured range is 2033 m. As can be seen in Figure 18b, a substantial portion of the turbine tower is occluded by the forest; only a small segment above the tree canopy, slightly above the rotor hub height, remains visible. The forest layer that occludes the turbine is not present in the echo plot because it is approximately 300 m from the radar site and outside the displayed range window. While a wind turbine may be considered quasi-stationary, a CAS radar is expected to detect it even under partial occlusion by intermediate objects.

As shown for ranges beyond 6 km, which are outside the primary scope of this work, we include results to demonstrate the radar’s ability to detect smaller dynamic targets. During the UAV tests at 6–7 km from the radar, the platform climbed from 300 m to 2000 m while flying circular patterns of 500 m radius at approximately 50 m/s. Figure 18 presents the echo from the UAV. The peak-to-background level in the detection image is noticeably lower than in other examples in this paper, due to the smaller RCS of the object, the longer range and different radar settings (chirp length and gain).

**Figure 19:**
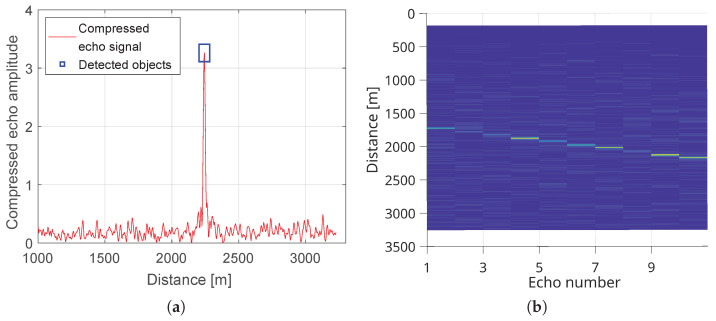
(**a**) Detection of a Cessna 172 at a range of 2243 m. (**b**) Range–time plot for ten detections of a Cessna 172. The color scale represents the normalized compressed echo amplitude over time and range, with brighter shades indicating stronger radar returns.

In the final case, we present tests with a Cessna 172 light aircraft. During the experiment, the aircraft approached along the LOS and then executed a turn, departing in the opposite direction. In Figure 19, a single detection at 2243 m is visible, together with ten range-time detections at 1 s intervals showing the aircraft receding from 1728 m to 2156 m.

An important point is to clarify the speed measurement capability of the system and the anti-collision response logic for dynamic targets. Radial speed is derived from range-time sequence processing by finite-difference smoothing of successive range estimates and optional local regression, yielding LOS range rate at the radar update rate. A kinematic tracker (α−β for quasi constant velocity segments, EKF for higher maneuverability [36]) fuses range and range-rate with bearing to produce a stabilized state vector. The EKF/UKF-based fusion with range and range-rate in cluttered radar scenarios is well documented in the literature. Alternatively, modern detector–tracker pipelines can be employed in real-time UAV stacks [37]. The anti-collision module continuously computes Time to Closest Point of Approach (TCPA) and predicted miss distance using short-horizon constant-velocity extrapolation. When TCPA falls below a preset bound and the miss distance violates the safety margin, the system issues graded responses: advisory alert, commanded lateral/vertical deviation, or loiter/braking. For small, highly maneuverable proximal hazards (e.g., birds), the short-range sensor layer supplies higher-rate updates to the same logic to ensure timely evasive action in cluttered environments.

## 6. Discussion

The obtained results confirm the high effectiveness of the applied detection approach based on a locally adaptive threshold maintaining a CFAR. Within the analyzed observation campaign, all objects visually identified in the co-registered daylight photographs were successfully detected by the radar system described in Section 3, indicating a clear one-to-one correspondence between the optical scene and the radar image. This finding is particularly significant in the context of low-altitude UAV operations, where reliable identification of obstacles in the 1–3 km range is a key factor for flight safety.

The spatial characteristics of echoes from objects (C), (D), and (E) are consistent with the expected mechanisms of electromagnetic wave scattering. The complex geometry of facades and mutual occlusion of buildings result in multiple secondary peaks, whose positions and amplitudes correspond well with the planar distribution of the structures. The analysis of inter-peak spacing and their stability across consecutive scans suggests that the radar system is capable not only of detecting the presence of objects but also of distinguishing architectural details such as recessed facades or protruding structural segments. This implies that the system can provide information that extends beyond classical point-obstacle detection, thereby supporting more detailed spatial scene modeling.

In Table 5, detections labeled as Unidentified were re-examined using field photographs and aerial images. Detection 10 is consistent with structural elements within object (E) (e.g., rooftop chimneys, antennas, facade recesses) and lies only 19 m from the dominant return of object (E), originating from features located near the building’s front. Detection 4 remains ambiguous and may originate from a temporary object (e.g., scaffolding, crane) or from multipath associated with objects (C) or (B), in the latter case it is treated by the CAS as a false-alarm candidate. Notably, detection 4 is located 37 m behind object (C), and because the system maintains a safety margin for obstacle depth, this ambiguity does not materially impact standoff decision-making.

It should be emphasized that the background level outside the detection regions remained low and spatially uniform, which indicates proper tuning of the CFAR algorithm. The absence of significant false returns further confirms the effectiveness of the thresholding method in suppressing noise and clutter while maintaining sensitivity to meaningful structural scatterers. However, this reconfigurable radar architecture enables the simple use of more refined detection algorithms, such as those presented in [38,39]. The strategy of excluding signals from the NR zone also proved justified-the echoes from close-in objects were effectively removed from the processing window, preventing receiver saturation and enhancing dynamic range utilization in the target interval. Diagnostic tests, in which the processing window was intentionally shifted earlier, confirmed the correctness of the timing budget and the proper functioning of the gating mechanism.

The comparison of radar-derived distances with GPS-based ground truth showed good agreement, with errors falling within the acceptable range for practical applications. The observed deviations can be primarily attributed to object geometry and inherent radar resolution limits. For the 11 detections with identified GPS ground truth, as presented in Table 4, the mean ranging error is 1.64 m and the root-mean-square error is 4.43 m, with the corresponding 95% confidence interval for the mean ranging error extending from −1.16 m to +4.43 m. These statistics indicate that the systematic bias of the radar remains small relative to the 3.75 m range resolution and to the safety margins typically adopted in CAS path-planning, and that the achieved accuracy is sufficient for use in CAS and UAV route planning in urban environments.

The proposed pulse radar prototype is considerably larger than the target form factor and is intended to validate functional correctness and the anticipated object-detection capability. To enable installation on an UAV of the intended class, miniaturization is required. As development progresses, successive subsystems are being reduced in size. At present, the RF front end relies on evaluation modules, and its power supply is provided by laboratory bench supplies.

In the first miniaturization phase, we aim to reduce the total system mass to below 2 kg. The system will include an antenna in two variants: 100 × 100 mm (120 g) and 200 × 200 mm (500 g), as well as an integrated digital board with the RF front end in a single enclosure with maximum dimensions of 100 × 200 × 50 mm and a mass up to 1.5 kg. While the PCB and antenna require only minor adjustments (e.g., connector and fastener reduction/replacement, removal of superfluous test components), the RF chain demands a thorough redesign. In subsequent steps, the team will focus on this task. Additionally, reducing the system’s power consumption is a key objective. This will be achieved by removing superfluous test circuitry, optimizing each of the TX, RX, and LO chains-particularly by refining PA control in the TX path-and by applying power-saving methods in the FPGA. We estimate that the average power draw in the default mode will decrease from 72 W to below 55 W.

One could consider abandoning the X-band in favor of the S-band (which is supported directly by the radar board). However, this would necessitate substantially larger antennas, or, if small antennas are retained, a degraded link budget coupled with a wider antenna beam. The latter negatively impacts the entire object detection methodology by reducing angular selectivity and increases clutter capture. This consideration is particularly critical for a stationary radar, which should detect objects aligned with the UAV’s flight path. The use of a gimbal is excluded due to the substantial increase in take-off mass. The situation differs for an AESA system: with electronic beam steering, it is straightforward to scan a wider volume with high precision. However, this advantage is practical only in the X-band (or higher bands), since an S-band AESA would require prohibitively large antenna aperture for the desired array size.

The team also evaluated the feasibility of implementing an AESA on the existing 200 × 200 mm antenna. The antenna’s structure allows the waveguide power-division network, visible in Figure 4, to be replaced with discrete TX/RX modules (i.e., physically independent transmit/receive channels with their own RF chains and control interfaces), enabling beamforming with four independent sub-arrays. This upgrade has a negligible impact on mass and power consumption. However, its cost is noticeably higher than a simpler, non-beam-steered solution. Nevertheless, such a configuration may appeal to more advanced platforms. Enabling electronic beam steering offers several benefits, notably direct angular information on targets, faster and more precise volume scanning, and adaptive sidelobe/clutter suppression; however, because only four ports are controlled in a 2 × 2 sub-array configuration, the achievable angular accuracy and beamforming granularity are inherently limited.

## 7. Conclusions

The lightweight pulse radar system presented in this work addresses important gaps in UAV collision avoidance systems by enabling reliable detection of urban obstacles at ranges of 1–3 km with $P_{\mathrm{FA}}$ of 1.5%, range resolution of 3.75 m, and maneuvering standoff times of 13 to 75 s for closing speeds between 40 and 80 m/s. These performance metrics considerably exceed the detection ranges of conventional FMCW radars limited to several hundred meters, thus enabling BVLOS operation for medium UAV platforms (10–300 kg MTOM).

Key innovations of the proposed system include integration of a single printed circuit board (60 g) employing IF transceiver and FPGA-based pulse compression algorithm with a fixed, forward-looking antenna, pioneering an X-band pulse radar architecture optimized for medium UAVs. The system offers runtime reconfigurability of radar parameters such as pulse repetition frequency, waveform chirp type, and adaptive CA-CFAR detection, which in field urban tests demonstrated a one-to-one correspondence between visual and radar-identified obstacles. A reduced electromagnetic footprint and average power consumption under 80 W (with development targets below 55 W) enhance operational endurance and electromagnetic compatibility in dense UAV traffic scenarios. Validation across multiple platforms, including Cessna 172 (2.2 km), Szogun UAV (6.4 km), and partially occluded wind turbines (2 km), confirms robustness across diverse urban targets and detection geometries, supporting deployment in complex operational environments where cognitive control architectures are essential [40].

The radar extends situational awareness for urban BVLOS flights by providing sufficient early-warning maneuver windows against static and dynamic obstacles such as buildings, towers, and other airspace users. Its range complements short-range sensors like LiDAR (effective typically below 1 km), supporting hybrid sensor fusion architectures essential for safe navigation at higher UAV speeds. Compliance with EASA C5/C6 certification categories and NATO STANAG 4671 enhances applicability in regulated airspace, while integration with extended Kalman filter-based tracking and TCPA algorithms supports efficient autonomous collision avoidance.

This development paves the way for scaling pulse radar technology via AESA implementations based on the modular antenna architecture (200 × 200 mm aperture with 27.4 dBi gain). Future improvements include driven target classification and multi-modal sensor fusion. Ongoing miniaturization efforts aim to achieve final system weights below 2 kg and power below 55 W, facilitating commercial deployment as UAV traffic density grows. Such a solution will significantly enhance the safety of BVLOS operations. These contributions position lightweight pulse radar as a cornerstone technology for scalable, robust collision avoidance systems enabling safe expansion of low-altitude UAV operations.

## Figures and Tables

**Table 1:** Comparison of recent UAV radar systems.

Reference	Radar Type	Frequency Band	Detection Range	Range Resolution	Power Consumption	Size/Weight
Our Radar	Pulse	X-band (9.5 GHz)	6.5 km (3.15 m span UAV)	3.75 m	80 W	Prototype (UAV-size under development)
[21]	FMCW	C/S-band	<1 km	∼1 m	N/A	N/A
[22]	FMCW	X-band (8.75 GHz)	∼2 km	∼0.5–1 m	N/A	Prototype
[23]	FMCW	W-band (77 GHz)	<0.5 km	N/A	Few W	Small
Commercial solution [24]	N/A	K-band (24 GHz)	6 km 2 km (Cessna)	N/A	45 W	19 × 12 × 4 cm, 820 g

**Table 2:** Default Parameters.

Parameter	Value
RF	9.5 GHz
RF power	43 dBm
RF bandwidth	40 MHz
IF	1.2 GHz
IF power	−10 dBm
IF bandwidth	20 MHz
BB $f_{s}$	40 MHz
BB bandwidth	10 MHz
BB pulse type	linear chirp
Weighting window	rectangular
PRF	10 kHz
Averaging rate	4
Min. Range	200 m
Max. Range ^1^	15,000 m
Pulse duration	2.5 μs
Duty cycle	2.5%
RX window length ^2^	7500 m
BB & IF processing power consumption	12 W
RF Front-end power consumption	60 W

^1^ The maximum range is determined by the PRF parameter. The effective radar range is shorter and defined by the RX window length, near-range gating, and pulse duration. ^2^ Window length expressed in meters. The corresponding window length in time is 50 μs.

**Table 3:** Configurable Parameters.

Parameter	Value
RF configuration	depends on RF Front-End
IF	0.2–3.6 GHz
IF power	−50–0 dBm
IF bandwidth	0–96 MHz
BB bandwidth	0.1–40 MHz
BB pulse type	chirp (linear, logarithmic), or custom signal
Weighting window	custom (programmable)
Max. PRF ^1^	50 kHz
Pulse duration	1–5 μs
Duty cycle	0.1–10%
Averaging rate	1–32

^1^ Determined by the DSP throughput. However, the maximum range and pulse duration parameters must be taken into account. A 50 kHz PRF corresponds to a maximum range of approximately 3 km, and the pulse duration should not exceed 5 μs.

**Table 4:** Characteristics of high buildings and landmarks within the radar field of view, including the object symbol, its description, distance from the radar to the object, height above ground level, and geographical coordinates.

Symbol	Description	Distance [m]	Height [m]	Geographical Coordinates
A	Terrace on the University’s D20 building	0	35.5	51.110146 N, 17.059800 E
	(radar location)			
NR	NR zone	0–800	5.0–25.0	—
B	Iglica	1128	90.3	51.107538 N, 17.075415 E
C	Centennial Hall (the dome)	1267	23.0	51.106922 N, 17.077213 E
D	Dormitory T-22 at Wittiga	1967	35.0	51.103938 N, 17.086182 E
E	Residential building at Kazimierska 21-17	2435	36.0	51.104320 N, 17.093426 E
F	Dormitory DS Arka	2778	35.5	51.102107 N, 17.097477 E
G	Dormitory DS-6 Raj	2948	32.0	51.101187 N, 17.099554 E

**Table 5:** List of radar detections as shown in Figure 12.

Detection Number	Description	Real (GPS) Distance [m]	Radar Distance [m]	GPS—Radar Difference ^1^ [m]
1	(B) Iglica	1128	1125	−3
2	Front wall of (C)	1241	1238	−3
3	(C) Centennial Hall (the dome)	1267	1268	+1
4	Unidentified object ^2^	—	1305	—
5	Two leftmost front buildings near (D)	1894	1890	−4
6	(D) Dormitory T-22 at Wittiga Street	1967	1969	+2
7	Rear protrusion of the first building near (E)	2375	2378	+3
8	Front wall of (E)	2427	2426	−1
9	(E) Residential building at Kazimierska 21-17	2435	2441	+6
	(top wall)			
10	Unidentified object ^3^	—	2460	—
11	Rear protrusion of the last building near (E)	2559	2561	+2
12	(F) Dormitory DS Arka	2778	2786	+8
13	(G) Dormitory DS-6 Raj	2948	2955	+7

^1^ Each entry in this column represents the ranging error for a single detection. For the 11 detections with identified GPS ground truth, the mean ranging error is 1.64 m, with a standard deviation of 4.16 m, a mean absolute error of 3.64 m, and a root-mean-square error of 4.43 m. The corresponding 95% confidence interval for the mean ranging error extends from −1.16 m to +4.43 m. ^2^ Detailed investigation did not identify any permanent object at this range. The source of this return may be a temporary structure (e.g., scaffolding, crane) or a multipath reflection. ^3^ Although the precise source of this return could not be uniquely determined, the evidence indicates that it originates from structures associated with building (E), located at a range of 2427–2484 m.

## Data Availability

All rights to the radar measured data are the exclusive property of EUROTECH Ltd.

## Abbreviations

The following abbreviations are used in this manuscript:

AESA	Active Electronically Scanned Array
AGL	Above Ground Level
BB	Base Band
BVLOS	Beyond Visual Line of Sight
CAS	Collision Avoidance System
CFAR	Constant False Alarm Rate
DSP	Digital Signal Processing
EASA	European Union Aviation Safety Agency
FMCW	Frequency-Modulated Continuous-Wave
FPGA	Field-Programmable Gate Array
GPS	Global Positioning System
IF	Intermediate Frequency
LiDAR	Light Detection and Ranging
LOS	Line of Sight
MIMO	Multiple-Input Multiple-Output
MTOM	Maximum Take-off Mass
NR	Near Range
OBC	On-Board Computer
PCB	Printed Circuit Board
$P_{\mathrm{FA}}$	Probability of False Alarm
$P_{D}$	Probability of target Detection
PRF	Pulse Repetition Frequency
RCS	Radar Cross-Section
RF	Radio Frequency
RX	Receiver
TCPA	Time to Closest Point of Approach
TX	Transmitter
UAV	Unmanned Aerial Vehicle
